# The Use of Selenium Yeast and Phytobiotic in Improving the Quality of Broiler Chicken Meat

**DOI:** 10.3390/foods10112558

**Published:** 2021-10-23

**Authors:** Damian Konkol, Małgorzata Korzeniowska, Henryk Różański, Wanda Górniak, Marita Andrys, Sebastian Opaliński, Ewa Popiela, Mariusz Korczyński

**Affiliations:** 1 Department of Animal Nutrition and Feed Management, Wrocław University of Environmental and Life Sciences, Chełmońskiego 38C, 51-630 Wrocław, Poland; damian.konkol@upwr.edu.pl (D.K.); mariusz.korczynski@upwr.edu.pl (M.K.); 2 Department of Functional Food Products Development, Wrocław University of Environmental and Life Sciences, Chełmońskiego 37, 51-630 Wrocław, Poland; malgorzata.korzeniowska@upwr.edu.pl; 3 Institute of Health and Economy, Carpathian State College in Krosno, Rynek 1, 38-400 Krosno, Poland; henryk.rozanski@adifeed.pl; 4 Department of Environment Hygiene and Animal Welfare, Wrocław University of Environmental and Life Sciences, Chełmońskiego 38C, 51-630 Wrocław, Poland; wanda.gorniak@upwr.edu.pl (W.G.); marita.swiniarska@upwr.edu.pl (M.A.); sebastian.opalinski@upwr.edu.pl (S.O.)

**Keywords:** selenium, meat quality, phytoncides, broilers, water-holding capacity, oxidative changes

## Abstract

The aim of the present study was to investigate the effect of selenium yeast and phytobiotic on the storage capacity, selected quality parameters of meat and content of selenium in muscles obtained from broilers. In the experiment, 1440 male broiler chickens (Ross 308) were randomly assigned to four research groups: group received no additive (G1), group received a supplement of 0.3 mg Se (as sodium selenite)/kg of feed mixture (G2), group received 0.2 g phytobiotic and 0.3 mg Se as 0.1 g selenium yeast per 1 kg of feed mixture (G3) and group received 0.3 mg Se as 0.1 g selenium yeast per 1 kg of feed mixture (G4). Measurement of pH, determination of water retention capacity, degree of advancement of oxidative changes and selenium content in muscles were performed. Samples of chickens’ breast and thigh muscles were microbiologically analyzed. The additives significantly influenced the level of oxidation in muscles and the incorporation of selenium. The meat of chickens receiving organic selenium was characterized by significantly lower dynamics of oxidative changes. The studies carried out showed that selenium in organic form had better absorption.

## 1. Introduction

Selenium is an essential element of human and animal diets due to the fact that it forms part of at least 25 proteins that play an important role in the regulation of homeostasis (e.g., glutathione peroxidase) [1,2]. Appropriate levels of selenium also determine the reproductive performance of animals, bone metabolism, immunological functions and iodine metabolism [3,4,5,6].

Feed selenium is available in various forms: inorganic types such as sodium selenite and organic types, e.g., selenium-enriched yeast, in which it is incorporated into certain peptides and amino acids such as selenomethionine, selenocysteine or selenocysteine. A similar form of selenium is present, for example, in cereal grains, and this form of selenium is best used by animals, which results from the fact that it is more active, less toxic and has higher bioavailability. Organic forms of selenium are usually used in the form of selenium yeast, chelates, proteinates, pure selenomethionine and 2-hydroxy-4methylselenobutanoic acid [7,8]. Currently, we can observe the increasing use of selenium nanoparticles in animal nutrition. Numerous studies have shown that nanoparticles of inorganic selenium are characterized by similar assimilation to organic forms of selenium, as well as significantly lower toxicity than inorganic forms, such as sodium selenite [9,10,11].

A group of substances that can also be used as feed additives are phytobiotics. Phytobiotics contain in their composition phytoncides, which are natural volatile compounds produced by plants for protection against pests. They are secondary metabolites of photosynthesis, enriched in terpenoids, phenylpropanoids and alkaloids. These compounds also inhibit the activity of microorganisms such as bacteria and fungi, so they can be good alternatives to feed antibiotics. Some phytogenic feed additives improve animal production parameters, feed–conversion ratio and animal carcass quality [12]. Essential oils contained in such additives have antioxidant properties [13]. By stimulating digestive enzymes, phytoncides improve the use of feed ingredients and affect gastrointestinal morphology [14].

Based on the available literature, it can be assumed that organic forms of selenium are better absorbed than inorganic forms [15,16]. Therefore, supplementation of organic selenium in broiler diets should improve the content of this element in the muscles of birds. On the other hand, the strong antioxidant properties of selenium should have an influence on better meat quality and extend its freshness. Moreover, the research hypothesis assumes that the absorption and properties of selenium should also be increased by the action of phytobiotic ingredients, which may enhance the antioxidant effect of selenium. Some studies showed that bioactive contents in these ingredients can play an important role in increasing the production of antioxidant enzymes such as glutathione peroxidase, which contains selenium [17].

The aim of the present study was to investigate the effect of selenium yeast belonging to the *Saccharomyces cerevisiae* strain NCYC R397 and phytobiotics (pepper dry fruit, cultivated mustard grain, extracts from common soapwort and sweet flag) on the storage capacity and some quality parameters of meat obtained from broiler chickens, as well as the content of selenium in the muscles of the tested animals. The use of this diet supplements combination can improve the bioavailability of selenium by supporting immune processes and stimulating digestive system functions, which will allow more effective use of the ingredients provided in the diet and achieve better meat properties.

## 2. Materials and Methods

### 2.1. Animals, Diets and Housing 

The research was carried out in the experimental henhouse of the Department of Environment, Hygiene and Animal Welfare at the Agricultural Experimental Plant “Swojec” in Wroclaw (Wroclaw, Poland). The experiment was carried out on Ross 308 broilers (males). The birds were kept in a deep litter system. The day-old chickens (1440) were placed in 24 boxes of 4 m^2^ each. The 24 boxes were divided into 4 research groups of 6 boxes (replications) for each of them. Each replication consisted of 60 chickens: group received no additive (G1), group received a supplement of 0.3 mg Se as sodium selenite (Sigma-Aldrich, Saint Louis, MO, USA)/kg of feed mixture (G2), group received 0.2 g phytobiotic and 0.3 mg Se as 0.1 g selenium yeast per 1 kg of feed mixture (G3) and group received 0.3 mg Se as 0.1 g selenium yeast per 1 kg of feed mixture (G4). The birds were fed in this way for 37 days. Selenium yeast belonging to the *Saccharomyces cerevisiae* strain NCYC R397 consists of approximately 65% selenomethionine, 17% selenocysteine, 5% unidentified selenium source, 11% water-soluble selenometabolites and 3% water-insoluble selenometabolites [18]. The phytobiotic (Adifeed, Warsaw, Poland) consisted of 0.5% hydrogenated palm oil, 98% herbs (pepper dry fruit, cultivated mustard grain, common soapwort, sweet flag), 0.5% thymol and nutritional additives and 1% ferric oxide. The birds were kept under controlled microclimatic conditions, according to the line manufacturer’s recommendations. The setpoint for temperature was 32 °C for Day 1; after that, it was slowly reduced to 22 °C till Day 21 and remained constant until Day 35. Relative humidity throughout the experiment was in the range of 50–60%. During the first week of the birds’ lives, the light day was interrupted every 6 h by 10 min periods of darkness. Thereafter, the light day was 18 h, and the dark period was 6 h, except for the last 3 days, during which the light was provided for 24 h. The average percentage of chicken mortality, throughout the experiment and for all groups, was 3.96%. Broiler chickens were fed according to the Poultry Nutrition Standards [19]. The feed used in the experiment was isoenergetic. After mixing, feed samples were taken, and the basic nutrients (dry matter, ash, crude protein, crude fiber, crude fat) were determined according to Wendee proximate analysis, as described by the AOAC 2016 procedure [20], while metabolic energy was determined using a calorimetric bomb in the laboratory of the Department of Animal Nutrition and Feed Management at the University of Environmental and Life Sciences. The composition of a starter and a grower mix is shown in Table 1, the chemical composition in Table 2 and the content of biologically active ingredients contained in the phytoncide preparation in Table 3.

### 2.2. Sample Collection and Muscles Analysis 

At the end of the feeding experiment on the 37th day, 7 random chickens were selected from each research group. In accordance with the Directive 2010/63/EU of the European Parliament and of the Council of 22 September, 2010, on the protection of animals used for scientific purposes [21], the chickens were killed by a percussive blow to the head and then exsanguinated. After exsanguination, the breast (*musculus pectoralis superficialis*) and thigh (*musculus iliotibialis lateralis*, *musculus femorotibialis lateralis*) muscles were removed from the carcass and further analyzed. Muscles were removed 10 min after slaughter, chilled in polythene bags on ice then frozen at −20 °C. This took place in the dissecting room within the experimental station where the experiment was performed. pH measurements were taken in ground meat samples 24 h, 5 and 7 days after slaughter by inserting a pH meter electrode (Orion 3-Star pH Benchtop Meter, Thermo Fisher Scientific Inc., calibrated on standards solutions with set pH). The water-holding capacity (WHC) of meat samples was analyzed according to the Grau–Hamm method with modifications by Szmańko [22]. Aliquots of meat samples were placed on Whatman No. 1 filter paper and pressed with a 2 kg metal block for 5 min. Cooking was subsequently conducted. The meat samples, packaged in cook-in plastic bags, were cooked in hot water at temperatures of 85 °C or 95 °C (hot water). All meat samples were heated until their geometric center temperature reached 72 °C, which was monitored by a thermocouple. The amount of water remaining after cooking relative to the water content in the original samples (without press and cooking) was taken as a measure of WHC. The degree of advancement of oxidative changes was carried out by the thiobarbituric acid method TBA according to the modified method of McDonald and Hultin [23] 24 h, 5 and 7 days after slaughter in meat samples, which, 10 min after slaughter, were chilled in polythene bags on ice then frozen at −20 °C. The raw breast and thigh muscles were ground in a meat grinder, homogenized and 0.5 g of the material was taken and mixed with 2 mL of 10% trichloroacetic acid (TCA). The suspension was centrifuged for 10 min at 4000× *g* (Sigma 3K30 Polygen). Next, 2 mL of 0.02 M thiobarbituric acid (TBA) was added to 2 mL of the collected supernatant and stirred vigorously. The samples were incubated at 100 °C in a water bath (Julabo EcoTemp TW 12) for 40 min. After 20 min of cooling under tap water, the absorbance was read against the blank (distilled water) at *λ* = 530 nm (Evolution 160 UV-VIS Thermo Scientific spectrophotometer). The results were calculated using the standard calibration curve based on the concentration of malonic dialdehyde (1,1,3,3-tetramethoxypropane) and expressed as mg of MDA per kg of meat. The procedure was carried out in triplicate. To determine the content of selenium, the muscles were freeze-dried, and the lyophilisates were digested in 65% nitric acid. Such digested samples were mineralized in a microwave mineralizer (MARS Extraction, MARSXpress Technology Inside, CEM Corporation). After evaporation, the samples were placed in an atomic absorption spectrometer (AA 240 FS, Fast Sequential Atomic Absorption Spectrometer, VARIAN). A flame atomizer (a mixture of air and acetylene) was used, and the carrier gas was argon, using the hydride generation method. The selenium was analyzed by measuring the absorbance peak area at 196 nm for each sample with background correction enabled. Background correction was performed using SpectrAA ver. 5.1 PRO software (Agilent, Santa Clara, CA, USA). The standard solution at various concentrations was used to construct standard curves for the assay [24]. All determinations were carried out in four replications and individual values were averaged. All the above analyses were performed for both the breast and thigh muscles. Samples of chicken breast and thigh muscles were microbiologically analyzed through the pour plate method in terms of the total number of microorganisms (according to ISO 4833-1: 2013-12) [25] Samples were homogenized with sterile peptone water (1.0 g/L peptone, 8.5 g/L NaCl) for 1 min with a laboratory blender (Easy Mix, AES Laboratories, Combourg, France). Series of dilutions were transferred into Petri dishes, then the culture medium (5.0 g/L tryptone, 2.5 g/L yeast extract, 12.0 g/L agar, BTL Ltd., Lodz, Poland) and 1.0 g/L anhydrous glucose (Chempur, Piekary Śląskie, Poland) were added to each plate and mixed carefully. Plates were incubated at 30 ± 1 °C for 72 h. Colonies of microorganisms were calculated using a hand-type colony counter (N.USUI & Co., Ltd., BIO KOBE, Japan) and expressed as total amount of microorganisms in 1 g of sample, *Enterobacteriaceae* (ISO 21528-2: 2005) [26] and *Campylobacter* spp. (ISO/TS 10272-2: 2008) [27]. The microbiological analysis was carried out on fresh material and material, which 10 min after slaughter, was chilled in polythene bags on ice, frozen at −20 °C and stored for 1, 6, 7 and 8 days. 

### 2.3. Statistical Analysis

Statistical analysis of the results was carried out using Statistica ver. 13.1. On the basis of the results obtained, for each parameter, we calculated the mean value. The standard error of measurement (SEM) and results were analyzed statistically:

- pH, TBA: analysis of diatomic variance, where differences between groups were assessed using the Tukey test;
- WHC and selenium content in muscles results by analysis of univariate variability and the significance of differences between groups using the Tukey test.

Differences were statistically significant when *p* < 0.05 or *p* < 0.01.

## 3. Results

The pH values of fresh muscles ranged between 6.16 and 6.33 and were generally higher than the mean values for white meat determined 24 h after slaughter (Table 4). Numerous cases of meat with metabolic defects were reported, which may be associated with preslaughter stress associated with a change in ambient temperature during transport to the place of slaughter. In general, minor changes were observed in birds from the G3 group. Storage of breast muscles for up to five days did not cause significant changes in the acidity of the meat. Further storage at refrigeration temperatures led to an increase in pH, which is related to the natural processes occurring in muscle tissue. The observed changes in pH did not indicate the initiation of the decay process. In the thigh muscles immediately after slaughter, the effect of dietary modifications on lower pH values in the G3 group was significant, indicating the beneficial effects of feed additives introduced during the rearing of birds.

Breast and thigh muscles of Ross 308 chickens analyzed immediately after slaughter were characterized by a low degree of fat oxidation (Table 5). There was no significant difference between the control group (negative control) and the experimental groups in the case of breast muscles. On the other hand, for thigh muscles, there were significantly smaller changes in lipids for the G4 group compared to the other groups. With the increase in storage time of both types of meat, the amount of dimalone aldehyde was increased, which is a measure of lipid oxidative changes. However, in the vast majority of cases, the values obtained are small and not indicative of meat degradation. The onset of meat degradation and the accompanying smell and rancid taste are well perceptible at TBA values higher than 4 mg of dimalone aldehyde per kg of meat. After 7 days, the lowest level of this parameter was observed in the G3 group, and this difference was significantly different in comparison to other groups. Analysis of variance of the obtained results showed significantly lower dynamics of oxidative changes in meat, especially thigh, obtained from chickens fed with selenium yeast. This is probably due to the strong antioxidant properties of selenium, which accumulated at greater levels in the meat of chickens fed its organic form.

Water-holding capacity for breast and thigh muscles was at an average of 57%. No significant effect of the additives on the ability of the meat components to retain water in their structures was observed. The content of selenium in the breast and thigh muscles presented very diverse levels (Table 6). The highest incorporation of selenium into the thigh muscles was obtained in chickens from the G3 and G4 groups. In the case of breast muscle, the highest selenium concentration was again observed in chickens from the G3 and G4 groups.

The total number of microorganisms, *Enterobacteriaceae* and *Campylobacter* spp. was determined for the breast and thighs muscles of the animals individually. These bacteria were measured for four random feeding groups and were measured during the first, sixth, seventh and eighth days. The sample taken during the first day was considered a reference level.

The results showed that for every feeding group and on each day of sample collection, the *Campylobacter* spp. number was less than 10 cfu/g. Facing the difference in several orders of magnitude compared with the other microorganisms amount, it was decided to exclude this from further deliberation.

**Figure 1:**
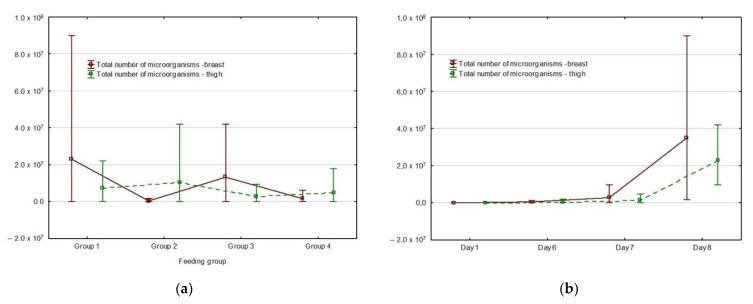

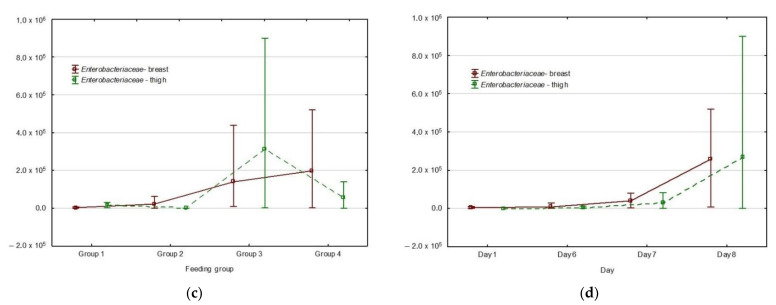
Mean plot of multiple variables grouped by day and feeding group; vertical lines show the nonoutlier range: (**a**) total number of microorganisms in the breast and thigh muscles relative to feeding groups; (**b**) total number of microorganisms in the breast and thigh muscles relative to day of the experiment; (**c**) *Enterobacteriaceae* in the breast and thigh muscles relative to feeding groups; (**d**) *Enterobacteriaceae* in the breast and thigh muscles relative to day of the experiment.

The total number of microorganisms in the breast and thigh muscles of the animals is depicted in Figure 1a. The total number of microorganisms in the breast and thigh muscles of the same animals is different, even though the feeding within the group was uniform. For example, the G1 group contained more bacteria in the breast muscles than in the thigh. Similarly, in the G2 group, the total number of microorganisms was negligible in the thigh, while the breasts contained a higher number of microorganisms. The G4 group contained the lowest amount of microorganisms in both the breast and thigh parts.

The total number of microorganisms did not change through the first week of the experiment (Figure 1b). A significant increase in the total number of microorganisms was observed on the last day of the experiment both in the case of breast and thigh muscles. 

Narrowing the bacteria type to *Enterobacteriaceae*, it can be seen that the G3 group contained the highest amount of such a microorganism in the thigh muscle (Figure 1c). An opposite tendency was presented in the breast muscle, where subsequent groups contained more *Enterobacteriaceae*. Furthermore, the obtained results show that on the last day of the experiment, the highest total number of microorganisms was noted, while there was almost no change within the first 7 days of the tests (see Figure 1d). 

## 4. Discussion

The cessation of blood circulation in the muscles after the slaughter of animals leads to a high accumulation of lactic acid, thus reducing the pH. A delayed drop in pH leads to a decrease in protein denaturation, thereby improving the muscle’s ability to retain water [28]. The results obtained in the course of our research did not show any significant influence of applied additives on the acidity of breast muscles of the tested birds. However, the present study showed significantly lower pH values in the case of thigh muscles from the group that received a supplement of phytobiotic and selenium yeast. It was also not shown whether the applied additives significantly affected water-holding capacity. The results obtained in this study are inconsistent with the results obtained by Wang et al. [16], where sodium selenite and two forms of selenomethionine were used as feed additives. Their study showed that the addition of selenomethionine significantly affected the pH of the breast muscles of the examined animals, and also, using different forms of selenomethionine in broiler chicken nutrition significantly reduced drip loss from the breast muscles of the examined animals. Another study conducted by Wang et al. [7] presented that the use of both sodium selenite and selenomethionine significantly influenced the breast muscle water-holding capacity of the tested chickens. Similar results were obtained by Bardzardi et al. [29], who reported that myrtle essential oil dietary supplementation had no effect on water-holding capacity. Additional results were also obtained by Li et al. [30], who found that the pH of the muscles obtained from chickens fed with selenium yeast, selenomethionine and nanoselenium was higher than that of the meat received from sodium selenite-fed chickens. Similar results were obtained by Mohammadi et al. [31], who used various forms of selenium in the feeding of broilers (sodium selenite, selenomethionine, nanoselenomethionine, Nano-Se-Max) and rosemary essential oil (REO). They showed no effect of the used additives on the pH of the muscles 4 h after slaughter. However, they presented that the pH of birds’ muscles that had not received selenium supplementation or REO tended to increase. They also found that REO significantly (*p* < 0.05) lowered the pH value of breast muscles in broiler chickens.

Antioxidant capacity is an important determinant of animal health and the quality of animal products. Dimalone aldehyde is one of the metabolic products of lipid peroxides and proteins and is widely used as a biomarker for oxidative stress [32]. The addition of selenium yeast increased the antioxidant capacity in the muscles of broiler chickens by reducing the production of oxidation products. These results are consistent with the results obtained by Wang et al. [7], which demonstrated that selenium supplementation in broiler chicken diet significantly increased total antioxidant capacity in the serum, liver, kidney and breast muscle of test subjects, with selenomethionine supplementation being more effective than sodium selenite supplementation. This study also demonstrated that selenomethionine supplementation significantly reduces the concentration of dimalone aldehyde in the test tissues. Slightly different results were obtained by Rozbicka-Wieczorek et al. [33], who showed that the addition of selenium (both inorganic and organic) to the broilers diet significantly reduced the content of dimalone aldehyde in the breast muscles of the studied birds. Differences in the results (significant effect of the inorganic form of selenium) may be due to the fact that selenium was given together with other additives (fish oil, lycopene). Other results were obtained by Li et al. [30], who found that the addition of selenium yeast, selenomethionine or nanoselenium did not affect the concentration of dimalonic aldehyde in the muscles of the studied birds. Mohammadi et al. [31] also did not find that the addition of selenium in various forms or REO influenced the concentration of dimalonic aldehyde in the breast muscles of the tested birds after 3 and 6 days of storage. They found, however, that after 9 days of storage, the muscles of birds that received sodium selenite, Nano-Se-Max, selenomethionine and mixtures of REO and selenomethionine were characterized by a lower level of dimalonic aldehyde. After 12 days of storage, the level of this aldehyde was significantly (*p* < 0.05) lower in the selenomethionine group, the mixture of REO and nanoselenomethionine and the mixture of REO and sodium selenite.

Selenium is an essential trace element in both animal and human diets. It is indispensable for the escalation of type I iodothyronine deiodinase and selenoprotein P, which play a key role in the production of thyroid hormones and selenium transgenes [34]. It is generally believed that organic forms of selenium are characterized by a better bioavailability and tissue retention rate than inorganic forms. Therefore, the accumulation of selenium in tissues is a very important criterion for the use of minerals [7,35]. The content of selenium in the muscles of the tested chickens confirmed that selenium in the organic form (selenium yeast) is better absorbed. Similar results were obtained by Markovic et al. [6], who showed that the level of selenium was significantly (*p* < 0.01) higher in the meat of birds receiving the selenium yeast additive compared to the control group (not receiving any supplement). Similar results were also obtained by Bardzardi et al. [29], showing that the highest concentration of selenium (*p* < 0.05) was characterized by breast muscles obtained from the groups receiving the addition of nano-selenomethionine. Bakhshalinejad et al. [36] also observed that the organic forms of selenium are better absorbed and significantly accumulate in the broiler muscles.

The results of the present study did not show any significant impact of the applied additives on the microbiological status of broiler chicken meat. This does not confirm the results obtained by Youssef et al. [37], who stated that the use of ginger extract in the nutrition of broilers significantly reduces the total number of bacteria and causes a slight decrease in *Staphylococcus aureus*, the total number of coliforms and coliforms from stool in fresh and frozen meat. Another study [38] also recorded that the addition of soy genistein and citrus hesperidin to the broiler diet positively impacts the microbial quality of broiler meat.

## 5. Conclusions

Based on the obtained results, it can be concluded that the breast and thigh muscles from the group receiving the supplement of phytobiotic and organic forms of selenium were characterized by the lowest pH. Based on physicochemical changes, the use of selenium yeast contributes to reducing the intensity of oxidative stress in broiler chicken meat, especially in thigh muscles. It was also shown that the use of selenium yeast leads to an increase in the level of this element in the meat of chickens. The addition of phytobiotics to feed increased the absorption of selenium from this organic source. However, further research is needed to develop feed additives that not only improve meat quality but also such additives that will help to accumulate selenium in meat in quantities optimal for human health. Phytobiotics should also be further investigated for synergistic effects with minerals.

## Figures and Tables

**Table 1:** Share of individual feed components in the basal starter and grower mix.

Starter		Grower	
Component	Content (%)	Component	Content (%)
Wheat	44.681	Wheat below 11% TP	42.948
Soybean meal	22.560	Soybean meal	21.860
Maize	20	Maize	15
Fishmeal total protein	0.500	Wheat full of grain	5
Soybean oil	1.380	Soybean oil	1.5
Rapeseed oilcake	4	Rapeseed oilcake	6.6
Calcium phosphate	0.850	Calcium phosphate	0.4
Feed salt	0.204	Feed salt	0.2
Acidic calcium carbonate	0.096	Acidic calcium carbonate	0.174
Choline chloride 60%	0.100	Choline chloride 60%	0.1
Sacox 210	0.053	Sacox 210	0.053
L-lysine HCl	0.174	L-lysine HCl	0.156
L-threonine	0.126	L-threonine	0.132
RHODIMET AT88	0.336	RHODIMET AT88	0.312
Fodder meal	0.780	Fodder meal	0.74
Sodium sulfate	0.100	Lysine sulfate (55% L-lysine)	0.4
Lysine sulfate (55% L-lysine)	0.300	Pork fat	2.5
Hemoglobin-pork blood product	2.060	Medium-chain fatty acids	1.6
Medium-chain fatty acids	1.300	Rovabio Excel LC2	0.01
Rovabio Excel LC2	0.010	Quantum Blue 5L	0.02
Quantum Blue 5L	0.020	Max-Vit 0.25% Prestige *	0.295
Max-Vit 0.25% Prestige *	0.370		

* Vitamin–mineral premix provides, per kilogram of starter: Vitamin A 14,800 IU, vitamin D3 7400 IU, vitamin E 111 mg, vitamin K3 8.8 mg, vitamin B1 4.5 mg, vitamin B2 14.8 mg, vitamin B6 7.5 mg, vitamin B12 230 µg, biotin 14.8 mg, folic acid 3.3 mg, calcium pantothenate 21 mg, niacin 88 mg, iron 69.8 mg, zinc 147.6 mg, iodine 1.8 mg, manganese 221.5 mg, copper 39 mg, and per kg of grower: Vitamin A 11,800 IU, vitamin D3 5900 IU, vitamin E 88.5 mg, vitamin K3 7 mg, vitamin B1 3.5 mg, vitamin B2 11.8 mg, vitamin B6 6 mg, vitamin B12 188 µg, biotin 11.8 mg, folic acid 2.6 mg, calcium pantothenate 16 mg, niacin 65.6 mg, iron 57.7 mg, zinc 117.4 mg, iodine 1.4 mg, manganese 176.6 mg, copper 31 mg, Sacox 210—coccidiostat, RHODIMET AT88—hydroxy analog of DL-methionine in liquid form, Rovabio Excel LC2—enzyme product (xylanase and β-glucanase), Quantum Blue 5L—enzyme product (phytase).

**Table 2:** Marked composition of feed.

Group		Dry Matter (%)	Ash (%)	Crude Protein (%)	Crude Fiber (%)	Crude Fat (%)	Selenium (mg/kg)	Metabolic Energy (kcal)
G1	Starter	89.90	4.38	20.85	2.68	3.49	0.102	3000.78
G2		90.26	4.57	19.75	3.36	4.80	0.375	3000.02
G3		91.20	4.20	20.90	2.85	4.17	0.357	2999.85
G4		91.03	4.47	21.42	2.75	4.45	0.282	2999.48
G1	Grower	88.86	3.92	18.47	3.67	7.55	0.140	3148.75
G2		88.78	3.99	19.01	3.41	7.58	0.327	3148.91
G3		89.09	3.79	18.37	3.68	7.23	0.363	3149.01
G4		89.96	3.82	18.09	3.53	7.74	0.425	3149.32

**Table 3:** Content of the main groups of active substances in the phytobiotic.

Group of Active Compounds	Minimum Content in 1 kg of Preparation
Glucosinolates	2600 mg
Curcuminoids	1900 mg
Essential oils	14,950 mg
Phenols and polyphenols	4950 mg

**Table 4:** pH of breast and thigh muscle of fresh chickens and chickens refrigerated for up to seven days.

Description			Breast Muscles	SEM	*p*-Value	Thigh Muscles	SEM	*p*-Value
Group	G1		6.19 ^ab^	0.011	<0.050	6.33 ^a^	0.020	<0.050
	G2		6.22 ^b^	0.010		6.33 ^a^	0.021	
	G3		6.16 ^a^	0.011		6.27 ^b^	0.011	
	G4		6.19 ^ab^	0.001		6.31 ^ab^	0.010	
Day	Day 1		6.16 ^a^	0.012	<0.050	6.28 ^a^	0.012	<0.050
	Day 5		6.18 ^a^	0.011		6.28 ^a^	0.010	
	Day 7		6.23 ^b^	0.013		6.33 ^b^	0.011	
Interaction	G1	Day 1	6.17	0.020	0.470	6.30	0.031	0.810
	G1	Day 5	6.19	0.022		6.31	0.030	
	G1	Day 7	6.22	0.021		6.37	0.021	
	G2	Day 1	6.18	0.031		6.31	0.032	
	G2	Day 5	6.20	0.020		6.31	0.030	
	G2	Day 7	6.28	0.012		6.37	0.021	
	G3	Day 1	6.14	0.021		6.26	0.020	
	G3	Day 5	6.15	0.021		6.26	0.023	
	G3	Day 7	6.18	0.022		6.28	0.020	
	G4	Day 1	6.18	0.010		6.30	0.021	
	G4	Day 5	6.19	0.010		6.31	0.022	
	G4	Day 7	6.21	0.001		6.32	0.020	

SEM—standard error of mean, a, b …—significance of differences on the level *p* < 0.05, a, a, b, b…—no significant differences, G1—group received no additive, G2—group received a supplement of 0.3 mg Se (as sodium selenite)/kg of feed mixture, G3—group received 0.2 g phytobiotic and 0.3 mg Se as 0.1 g selenized yeast per 1 kg of feed mixture, G4—group received 0.3 mg Se as 0.1 g selenized yeast per 1 kg of feed mixture.

**Table 5:** Dimalone aldehyde (TBA) content in breast and thigh muscles in fresh chickens and chickens refrigerated for up to seven days (μg/g meat).

Description			Breast Muscles	SEM	*p*-Value	Thigh Muscles	SEM	*p*-Value
Group	G1		3.00 ^ab^	0.151	<0.050	3.28 ^a^	0.192	<0.050
	G2		2.87 ^a^	0.140		3.23 ^a^	0.163	
	G3		2.83 ^a^	0.122		3.37 ^a^	0.181	
	G4		3.17 ^b^	0.101		2.75 ^b^	0.120	
Day	Day 1		2.04 ^a^	0.040	<0.050	1.84 ^a^	0.040	<0.050
	Day 5		3.05 ^b^	0.052		3.59 ^b^	0.062	
	Day 7		3.88 ^c^	0.051		3.77 ^c^	0.061	
Interaction	G1	Day 1	1.89 ^a^	0.081	<0.050	1.71 ^a^	0.060	<0.050
	G1	Day 5	2.97 ^bcd^	0.143		4.12 ^def^	0.173	
	G1	Day 7	4.16 ^g^	0.092		4.02 ^ef^	0.181	
	G2	Day 1	1.92 ^a^	0.060		1.90 ^a^	0.122	
	G2	Day 5	2.65 ^bc^	0.071		3.92 ^cdef^	0.130	
	G2	Day 7	4.04 ^g^	0.081		3.86 ^cdef^	0.171	
	G3	Day 1	1.86 ^a^	0.090		1.85 ^a^	0.070	
	G3	Day 5	3.25 ^def^	0.113		4.01 ^def^	0.183	
	G3	Day 7	3.38 ^f^	0.121		4.25 ^f^	0.141	
	G4	Day 1	2.47 ^a^	0.062		1.87 ^a^	0.150	
	G4	Day 5	3.30 ^b^	0.110		3.10 ^b^	0.091	
	G4	Day 7	3.76 ^bc^	0.121		3.28 ^bc^	0.172	

SEM—standard error of mean, a, b, c, d, e, f, g …—significance of differences on the level *p* < 0.05, a, a, b, b, c, c, d, d, e, e, f, f, g, g…—no significant differences, G1—group received no additive, G2—group received a supplement of 0.3 mg Se (as sodium selenite)/kg of feed mixture, G3—group received 0.2 g phytobiotic and 0.3 mg Se as 0.1 g selenized yeast per 1 kg of feed mixture, G4—group received 0.3 mg Se as 0.1 g selenized yeast per 1 kg of feed mixture.

**Table 6:** Water-holding capacity and selenium content in the muscles.

Group	Selenium Content (μg/kg)		Water-Holding Capacity (%)					
	Thigh Muscles	Breast Muscles	Breast	SEM	*p*-Value	Thigh	SEM	*p*-Value
G1	248 ^A^	360 ^A^	57.6	0.06	0.100	57.6	0.07	0.100
G2	461 ^B^	406 ^A^	57.4	0.07		57.5	0.06	
G3	896 ^C^	708 ^B^	57.8	0.06		57.4	0.05	
G4	763 ^D^	733 ^C^	57.7	0.05		57.4	0.06	
SEM	36.8	27.5						
*p*-Value	0.001	0.001						

SEM—standard error of mean, A, B, C, D …—significance of differences on the level *p* < 0.01, A, A …—no significant differences, G1—group received no additive, G2—group received a supplement of 0.3 mg Se (as sodium selenite)/kg of feed mixture, G3—group received 0.2 g phytobiotic and 0.3 mg Se as 0.1 g selenized yeast per 1 kg of feed mixture, G4—group received 0.3 mg Se as 0.1 g selenized yeast per 1 kg of feed mixture.

## Data Availability

The data presented in this study are available on request from the corresponding author.
